# Zoom in at African country level: potential climate induced changes in areas of suitability for survival of malaria vectors

**DOI:** 10.1186/1476-072X-13-12

**Published:** 2014-05-07

**Authors:** Henri EZ Tonnang, David P Tchouassi, Henry S Juarez, Lilian K Igweta, Rousseau F Djouaka

**Affiliations:** 1 International Centre of Insect Physiology and Ecology (icipe), Nairobi, Kenya; 2 Agrosanidad SAC / Archana EIRL, Jerez Q-8 Mayorazgo III Etapa, Lima 03, Peru; 3 International Institute of Tropical Agriculture (IITA), Cotonou, Benin

**Keywords:** Climate change, Eco-climatic index, African countries, *Anopheles gambiae* s.s, *Anopheles arabiensis*

## Abstract

**Background:**

Predicting anopheles vectors’ population densities and boundary shifts is crucial in preparing for malaria risks and unanticipated outbreaks. Although shifts in the distribution and boundaries of the major malaria vectors (*Anopheles gambiae* s.s. and *An. arabiensis*) across Africa have been predicted, quantified areas of absolute change in zone of suitability for their survival have not been defined. In this study, we have quantified areas of absolute change conducive for the establishment and survival of these vectors, per African country, under two climate change scenarios and based on our findings, highlight practical measures for effective malaria control in the face of changing climatic patterns.

**Methods:**

We developed a model using CLIMEX simulation platform to estimate the potential geographical distribution and seasonal abundance of these malaria vectors in relation to climatic factors (temperature, rainfall and relative humidity). The model yielded an eco-climatic index (*EI*) describing the total favourable geographical locations for the species. The *EI* values were classified and exported to a GIS package. Using ArcGIS, the *EI* shape points were clipped to the extent of Africa and then converted to a raster layer using Inverse Distance Weighted (IDW) interpolation method. Generated maps were then transformed into polygon-based geo-referenced data set and their areas computed and expressed in square kilometers (km^2^).

**Results:**

Five classes of *EI* were derived indicating the level of survivorship of these malaria vectors. The proportion of areas increasing or decreasing in level of survival of these malaria vectors will be more pronounced in eastern and southern African countries than those in western Africa. Angola, Ethiopia, Kenya, Mozambique, Tanzania, South Africa and Zambia appear most likely to be affected in terms of absolute change of malaria vectors suitability zones under the selected climate change scenarios.

**Conclusion:**

The potential shifts of these malaria vectors have implications for human exposure to malaria, as recrudescence of the disease is likely to be recorded in several new areas and regions. Therefore, the need to develop, compile and share malaria preventive measures, which can be adapted to different climatic scenarios, remains crucial.

## Background

Efforts have been made to predict and map the potential redistribution of malaria vectors and consequently malaria transmission risk especially in the context of climate change. Such malaria risk projections invaluably may provide policy–makers with the opportunity to initiate activities for identifying vulnerable communities in a timely manner and to develop effective strategies to curtail and prevent malaria outbreaks [1,2]. Diverse models have been used in projection studies thereby producing different malaria risk projections and corresponding shifts in future distribution and variability of malaria transmission. Most models have however, focused on key climate-based parameters such as temperature, rainfall and relative humidity as these influence the breeding, emergence and abundance of *Anopheles* vectors and parasite development, which is correlated with increased biting and transmission of malaria [3-5].

Peterson [6] applied ecological niche model using Genetic Algorithm for Rule-set Prediction (GARP) [7] and predicted the potential distributional shift from west to east and west to south of Africa for *An. gambiae* s.s. and *An. arabiensis,* respectively. Within the same context [8] it has been suggested however, that *Anopheles* distribution range shifts are more likely to occur than range expansions. Moreover, several studies have suggested a high probability of distributional change of current regions where malaria vectors specially occur and may potentially occur in the future with climate change [3,6-14].

Shifts in malaria transmission risk have mainly been projected on a global or continental scale with little efforts within regional or local scales (i.e., country-specific) especially in malaria disease endemic countries of Africa. Projection of future malaria risks taking into account local climatic conditions has been emphasized [15-17]. Studies of climate–malaria relationships within local scales are likely to be more useful for public health officials than broad, global–scale studies. Specifically, finer–scale analyses facilitate incorporation of local features and characteristics and may provide a greater opportunity for intervention and response, given that public health programs are typically applied at the regional or local level [18,19].

In an earlier study [14], we used the software, CLIMEX, to calibrate the current and future distributions of key malaria vectors in Africa. Using this tool, the potential geographical distribution and seasonal abundance of the species in relation to climatic factors such as temperature, rainfall and relative humidity were estimated. As such, boundary shifts of the major Africa malaria vectors, *Anopheles gambiae* s.s*.* (herein referred to as *An. gambiae*) and *An. arabiensis* were predicted southward and eastward of Africa, respectively. Malaria vector control activities appear to be country specific in Africa and continental information as presented in Tonnang et al. [14] may either be neglected or not be applicable to every country, hence a country level projection required. Climate change potential inducing shifts of these vectors may have negative impacts for human exposure to malaria, as recrudescence of the disease is likely to be recorded in several new areas within a country. It is necessary to project how each country’s territory will be affected as a prerequisite to develop, compile and share malaria preventive measures, which can be adapted to different climatic scenarios. While acknowledging the impacts of climate change on the future distribution of these malaria vectors with consequent shifts in their overall ranges, the present study sought to quantify the areas of absolute change per country in Africa beyond the already projected shifts for these species.

## Methods

### Climate and malaria vectors data sets

The climate data used for spatial simulations (current scenario) were obtained from the Climatic Research Unit (CRU) in Norwich UK available at http://www.cru.uea.ac.uk/. The distributional records for *An. gambiae* and *An. arabiensis* used in this study were obtained from Mapping Malaria Risk in Africa (MARA) collaboration (http://www.mara.org.za/) repository of information [20]. This database contains 2,535 geo-referenced records of malaria vectors of Africa. Additional geo-reference information on malaria vectors was sourced from recent publications [21,22].

### Climate change projections

For predicting the future potential distributions of species, CLIMEX platform incorporates the IPCC climate change scenarios. It is projected that globally average surface temperature is to increase by 1.4 to 5.8°C and a considerable change in the pattern of rainfall will occur by the year 2100 [23,24]. Within the specified framework two projected scenarios were considered as described below:

– Scenario 1: A rise of 2°C Africa wide temperature and 10% increase of rainfall from March 2 -September 30 and 10% decrease in the rest of the year.

– Scenario 2: A rise of 0.1°C in the whole year maximum and minimum temperatures per degree of latitude, and a 10% increase of rainfall from March 2-September 30 and 10% decrease in the rest of the year.

### Model development

The simulation that yielded the eco-climatic index (*EI*) was conducted inductively with CLIMEX platform [23]. CLIMEX makes use of the species responses to a series of stress indices (cold, hot, dry and wet) that define the species’ particular environmental limits for a sustained growth and development of the population [23]. These stress indices were linearly combined to the species’ growth indices to yield an *EI* that describes the total favourability of geographic locations for the species [14,23]. In calibrating the malaria vectors models, the “compare locations” function was run. This function links the *EI* to the “match climate” function that compares the meteorological data from different places, with no reference to the preferences of *An. gambiae* and *An. arabiensis*, respectively, [14].

The malaria vector distribution shape points [14] were obtained with the following procedure: Firstly, best fitted CLIMEX parameters obtained through multiple run of the “*compare location*” function were used for mapping the *Anopheles* species under current scenario. Secondly, the “*compare location*” function was once again run with the best-matched CLIMEX parameters and the *EI* for the *An. gambiae* and *An. arabiensis* were estimated under the chosen projected scenarios. The results were then transferred to Arc-GIS v. 9.2 (ESRI) package in order to facilitate the organization, manipulation, visualization, geospatial analysis and absolute area computations.

### Evaluating and quantifying the absolute range change of An. gambiae and An. arabiensis

**Figure 1:**
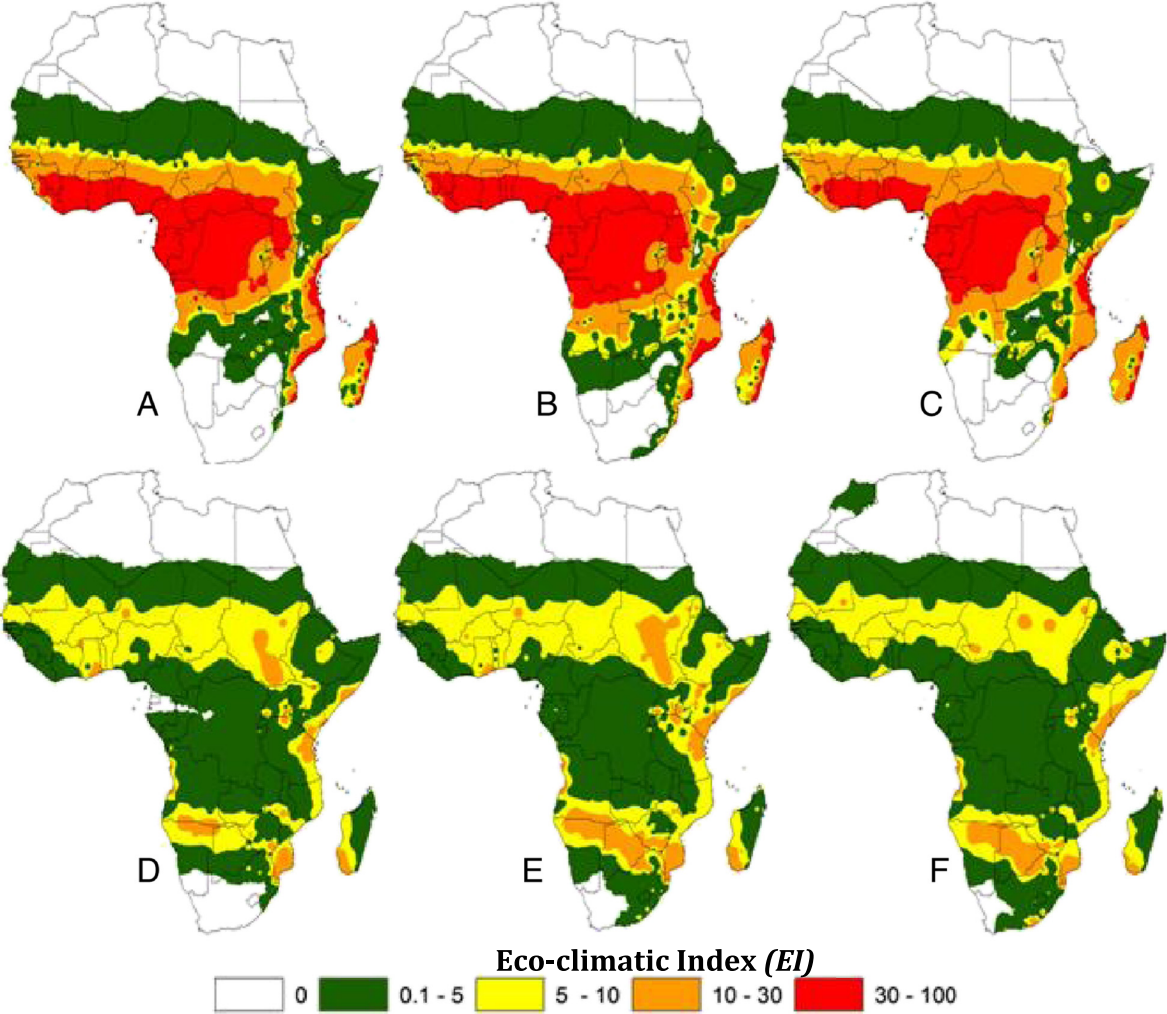
**Range shifts in the distribution of *Anopheles gambiae *(A, B, C) and *An. arabiensis *(D, E, F) using the Eco-climatic indices (*EI*).** Indices reclassified from the original data by Tonnang *et al.*[14]; **(A, D)**, Eco-climatic indices for current climate; **(B, E)** and **(C, F)** correspond to climate change scenario 1 and 2 respectively.

The CLIMEX parameters values were identical to the original model calibrated and validated for *An. gambiae* and *An. arabiensis* by Tonnang et al. [14]. The eco-climatic index (*EI*) represented in form of maps was also similar to the results published in Tonnang et al. [14]. With ArcGIS v. 9.2 (ESRI) the shape point’s outputs from CLIMEX for all scenarios (current *EI* and projected *EI* for scenarios 1 and 2) were clipped to the extent of Africa. Then, each point file was converted to a raster layer using Inverse Distance Weighted (IDW) interpolation method [25] with a cell size of 3 minutes in both longitude and latitude. The maps were reclassified (Figure 1) and used as the foundation for this study. Reclassification consisted of taking input cell values and replacing them with new output cell values. It was used to simplify the interpretation of raster data by changing a single value to a new value, or grouping ranges of values into single values. The operations merely repackage existing information on a single map. The reclassified maps were converted into a polygon-based geo-referenced data set for computing areas in square kilometers (km^2^) for the polygons. From the total polygon areas belonging to the same country, individual area was estimated. To estimate per country the absolute change of suitability, the difference in area between the values of *EI* for each projected scenario and the current scenario was performed for *An. gambiae* and *An. arabiensis*. Further, the percentage of range shift per country was calculated by dividing the area of absolute change over the total area of vector distribution for each country obtained from the current scenario.

## Results

**Figure 2:**
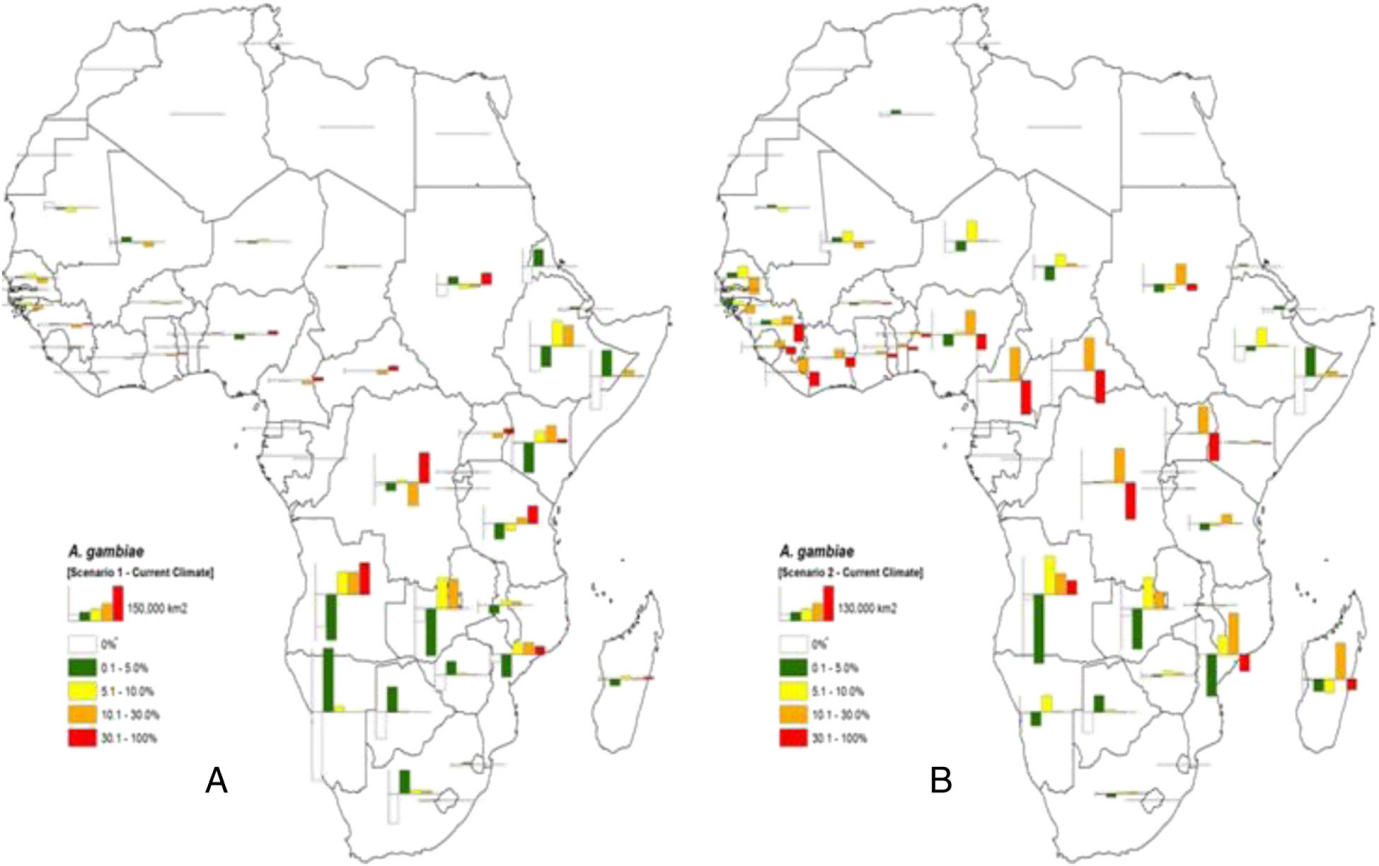
**Bar charts showing the absolute change in area (km**^**2**^**) that may occur due to possible change in climate for *Anopheles gambiae *per African country.** Values obtained by subtracting the Eco-climatic index (*EI*) estimates of projected scenarios 1 **(A)** and 2 **(B)** criteria to the *EI* estimates obtained under current climate for each country. The height of a bar shows the proportion of area in a scale of **(A)** 150,000 km^2^ and **(B)** 130,000 km^2^; bar display on the positive axis represents an increasing area and a reverse bar in the negative axis, a decreasing area.

Regions where the Eco-climatic index (*EI*) equals 100 indicate climate conditions where a certain proportion of the malaria vectors population is expected to survive throughout the year, representing the regions where the likelihood of establishment is highest. In zones where the *EI* deviates from the maximum number of 100, the likelihood of long-term survival is reduced. Based on this concept as well as referring to the actual known distributions of the species under investigation [11]; 5 classes of *EI* were considered as shown in Figure 2: Class 1 (*EI* = 0) indicates that the location is not suitable for the species. Class 2 (*EI* ≤ 5) represents zones with very little suitability for the survival of malaria vectors. Class 3 (*EI* = (5–10)) indicates areas with sequential risk of establishment of the malaria vectors. Class 4 (*EI* = (10–30)) indicates areas with high risk of permanent establishment of malaria vector. In Class 5 (*EI* > 30) the likelihood of long-term survival of *An. gambiae* and *An. arabiensis* is very high. Under projected scenarios 1 and 2, and for both malaria vectors, a country belonging to any class with the area equals to 0 is a territory with no range shift (Tables 1, 2, 3, 4). A negative value of an area signifies that a reduction of the area size will occur, whereas a positive value indicates an increment.

**Table 1:** **Absolute change in areas (km**^**2**^**) for *An. gambiae *** **per country of Africa under scenario 1**

**Countries**	***EI*** **= 0**		***EI*** **= (0.1-5)**		***EI*** **= (5–10)**		***EI*** **= (10–30)**		***EI*** **= (30–100)**	
	**(km**^**2**^**)**	**%**	**(km**^**2**^**)**	**%**	**(km**^**2**^**)**	**%**	**(km**^**2**^**)**	**%**	**(km**^**2**^**)**	**%**
Algeria	-268	0.0%	268	0.7%	0	--	0	--	0	--
Angola	-190,987	-98.3%	-269,996	-60.8%	137,090	117.6%	133,655	38.5%	190,238	126.3%
Benin	0	0.0%	0	--	0	--	-5,198	-9.5%	5,198	8.1%
Botswana	-156,215	-32.2%	151,820	160.1%	3,827	--	567	--	0	--
Burkina Faso	0	--	-25	-0.1%	5,389	12.7%	-7,498	-3.7%	2,134	45.0%
Burundi	0	--	-276	-32.1%	-540	-28.9%	-111	-0.5%	927	336.4%
Cameroon	0	0.0%	-2,418	-56.4%	1,511	19.0%	-18,380	-16.2%	19,287	5.7%
Cape Verde	0	0.0%	0	--	0	--	0	--	0	--
Central African Republic	0	--	0	--	0	--	-23,133	-20.5%	23,133	4.6%
Chad	-237	-0.7%	-9,195	-1.1%	2,241	1.6%	3,756	1.4%	3,435	--
Comoros	0	0.0%	0	--	0	--	176	402.2%	-176	-19.9%
Congo	0	0.0%	0	--	0	--	0	--	0	0.0%
Congo, DRC	-11,592	-98.8%	-47,801	-63.7%	13,711	33.2%	-134,908	-29.4%	180,590	10.3%
Cote d’Ivoire	0	0.0%	0	--	0	--	-3,511	-52.7%	3,511	1.1%
Djibouti	-13,032	-92.5%	13,032	203.0%	0	--	0	--	0	--
Egypt	0	0.0%	0	--	0	--	0	--	0	--
Equatorial Guinea	0	0.0%	0	--	0	--	0	--	0	0.0%
Eritrea	-99,158	-88.3%	99,158	1307.1%	0	--	0	--	0	--
Ethiopia	-151,552	-96.4%	-122,837	-14.7%	152,510	195.6%	121,818	189.4%	61	7.4%
Gabon	0	0.0%	0	--	0	--	0	--	0	0.0%
Ghana	0	0.0%	0	--	0	--	-7,835	-16.2%	7,835	4.1%
Guinea	61	8.4%	5,797	148.1%	4,051	69.2%	-17,185	-12.9%	7,276	7.1%
Guinea-Bissau	43	4.7%	6,578	--	15,656	--	-22,277	-73.1%	0	--
Kenya	-1,924	-7.2%	-179,902	-49.9%	64,693	82.0%	98,760	115.2%	18,373	55.6%
Lesotho	-2,282	-7.4%	2,282	--	0	--	0	--	0	--
Libya	0	0.0%	0	0.0%	0	--	0	--	0	--
Madagascar	-261	-4.7%	-37,823	-57.2%	18,757	15.8%	5,219	2.0%	14,108	10.4%
Malawi	-357	-54.6%	-47,014	-68.1%	27,654	98.1%	19,343	98.8%	373	--
Mali	-3,425	-1.4%	31,341	4.8%	-3,096	-2.2%	-25,456	-12.0%	636	60.8%
Mauritania	31,741	11.7%	-10,301	-1.4%	-21,440	-69.1%	0	--	0	--
Morocco	0	0.0%	0	--	0	--	0	--	0	--
Mozambique	-44,578	-87.9%	-136,287	-53.4%	65,073	66.7%	70,031	25.9%	45,761	37.9%
Namibia	-409,359	-54.1%	380,869	532.2%	27,946	--	544	--	0	--
Niger	-3,060	-2.9%	-9,587	-0.9%	12,169	27.3%	477	3.3%	0	--
Nigeria	0	0.0%	-28,855	-46.3%	5,777	3.6%	1,169	0.4%	21,909	5.8%
Rwanda	0	--	-518	-28.6%	-3,817	-48.5%	4,335	28.2%	0	--
Sao Tome & Principe	0	0.0%	0	--	0	--	0	--	0	0.0%
Senegal	0	0.0%	8,049	20.0%	19,902	36.5%	-27,951	-27.4%	0	--
Seychelles	0	0.0%	0	--	0	--	0	--	0	--
Sierra Leone	0	0.0%	0	0.0%	0	0.0%	-1,924	-6.7%	1,924	5.0%
Somalia	-198,568	-91.2%	155,491	52.5%	4,854	7.1%	34,807	62.5%	3,416	--
South Africa	-176,144	-14.8%	139,066	512.6%	20,468	739.0%	14,615	662.5%	1,995	--
Sudan	-68,926	-10.4%	44,233	5.4%	-24,274	-11.7%	-17,704	-2.8%	66,671	37.6%
Swaziland	-11,056	-77.9%	9,423	357.2%	1,524	--	108	--	0	--
Tanzania	-1,477	-30.1%	-93,706	-45.6%	-43,689	-24.8%	34,179	8.3%	104,692	71.4%
The Gambia	0	0.0%	0	--	70	--	-70	-0.7%	0	--
Togo	0	0.0%	0	--	0	--	-1,293	-12.3%	1,293	2.8%
Tunisia	0	0.0%	0	--	0	--	0	--	0	--
Uganda	0	0.0%	-383	-20.1%	-519	-24.8%	-24,531	-41.2%	25,433	14.0%
Western Sahara	410	0.2%	-410	-0.6%	0	--	0	--	0	--
Zambia	-75,481	-96.6%	-280,531	-45.0%	182,310	385.4%	173,703	3221.2%	0	--
Zimbabwe	-90,517	-37.7%	79,923	57.9%	8,760	65.2%	1,834	--	0	--
**Grand total**	**-1,678,200**	**-15.9%**	**-150,534**	**-1.9%**	**698,477**	**40.6%**	**378,946**	**8.6%**	**751,311**	**13.9%**

Values obtained by subtracting the Eco-climatic index (*EI*) estimates for future *An. gambiae* distribution from projected scenario 1 criteria to the *EI* estimates obtained under current climate for each country. Percentages (%) were calculated by dividing each absolute area change to the current area value per country.

**Table 2:** **Absolute change in areas (km**^**2**^**) for *An. gambiae *** **per country of Africa under scenario 2**

**Countries**	***EI*** **= 0**		***EI*** **= (0.1-5)**		***EI*** **= (5–10)**		***EI*** **= (10–30)**		***EI*** **= (30–100)**	
	**(km**^**2**^**)**	**%**	**(km**^**2**^**)**	**%**	**(km**^**2**^**)**	**%**	**(km**^**2**^**)**	**%**	**(km**^**2**^**)**	**%**
Algeria	-13,395	-0.6%	13,395	35.7%	0	--	0	--	0	--
Angola	-18,304	-9.4%	-262,407	-59.0%	144,986	124.4%	81,332	23.4%	54,392	36.1%
Benin	0	0.0%	0	--	0	--	9,805	18.0%	-9,805	-15.4%
Botswana	-76,585	-15.8%	64,040	67.5%	11,447	--	1,098	--	0	--
Burkina Faso	0	--	-7,385	-28.2%	6,067	14.3%	5,584	2.8%	-4,266	-90.0%
Burundi	0	--	31	3.6%	380	20.3%	-135	-0.6%	-276	-100.0%
Cameroon	31	5.5%	-2,620	-61.2%	4,795	60.2%	126,511	111.2%	-128,716	-37.9%
Cape Verde	0	0.0%	0	--	0	--	0	--	0	--
Central African Republic	0	--	0	--	0	--	124,589	110.5%	-124,589	-24.6%
Chad	-1,036	-3.0%	-52,916	-6.4%	43,927	30.9%	10,026	3.7%	0	--
Comoros	0	0.0%	0	--	0	--	840	1915.1%	-840	-94.7%
Congo	0	0.0%	0	--	0	--	0	--	0	0.0%
Congo, DRC	-1,022	-8.7%	2,929	3.9%	7,064	17.1%	131,275	28.6%	-140,247	-8.0%
Cote d’Ivoire	0	0.0%	0	--	2,002	--	32,490	487.5%	-34,492	-11.0%
Djibouti	-13,118	-93.1%	13,118	204.3%	0	--	0	--	0	--
Egypt	0	0.0%	0	--	0	--	0	--	0	--
Equatorial Guinea	0	0.0%	0	--	0	--	0	--	0	0.0%
Eritrea	-6,757	-6.0%	6,757	89.1%	0	--	0	--	0	--
Ethiopia	-56,353	-35.8%	-18,488	-2.2%	69,136	88.7%	6,531	10.2%	-826	-100.0%
Gabon	0	0.0%	0	--	0	--	0	--	0	0.0%
Ghana	0	0.0%	0	--	0	--	12,508	25.9%	-12,508	-6.5%
Guinea	90	12.5%	16,541	422.6%	17,106	292.4%	32,242	24.2%	-65,979	-64.8%
Guinea-Bissau	124	13.3%	17,947	--	12,398	--	-30,469	-100.0%	0	--
Kenya	-896	-3.4%	3,260	0.9%	-4,980	-6.3%	7,993	9.3%	-5,376	-16.3%
Lesotho	0	0.0%	0	--	0	--	0	--	0	--
Libya	0	0.0%	0	0.0%	0	--	0	--	0	--
Madagascar	-394	-7.0%	-46,954	-71.0%	-50,862	-42.8%	138,412	52.1%	-40,203	-29.7%
Malawi	30	4.5%	6,601	9.6%	-3,273	-11.6%	-3,357	-17.2%	0	--
Mali	-35,762	-14.2%	17,630	2.7%	41,122	28.9%	-21,945	-10.3%	-1,046	-100.0%
Mauritania	1,426	0.5%	10,121	1.4%	-11,546	-37.2%	0	--	0	--
Morocco	0	0.0%	0	--	0	--	0	--	0	--
Mozambique	-496	-1.0%	-161,657	-63.4%	70,282	72.1%	156,528	58.0%	-64,656	-53.5%
Namibia	-8,608	-1.1%	-54,034	-75.5%	60,700	--	1,942	--	0	--
Niger	-41,835	-40.1%	-37,796	-3.7%	77,389	173.6%	2,242	15.5%	0	--
Nigeria	0	0.0%	-43,815	-70.3%	10,834	6.8%	90,909	29.3%	-57,928	-15.2%
Rwanda	0	--	335	18.5%	908	11.5%	-1,244	-8.1%	0	--
Sao Tome & Principe	0	0.0%	0	--	0	--	0	--	0	0.0%
Senegal	0	0.0%	20,008	49.8%	42,788	78.5%	-62,796	-61.5%	0	--
Seychelles	0	0.0%	0	--	0	--	0	--	0	--
Sierra Leone	0	0.0%	1,852	91.8%	2,736	96.9%	21,979	76.0%	-26,568	-68.6%
Somalia	-144,129	-66.2%	113,211	38.2%	8,157	12.0%	19,397	34.8%	3,364	--
South Africa	-211	0.0%	-13,589	-50.1%	5,350	193.1%	6,716	304.4%	1,735	--
Sudan	-6,670	-1.0%	-30,065	-3.6%	-17,980	-8.7%	77,773	12.4%	-23,059	-13.0%
Swaziland	-404	-2.8%	55	2.1%	350	--	0	--	0	--
Tanzania	-184	-3.7%	-25,898	-12.6%	-8,435	-4.8%	34,099	8.3%	416	0.3%
The Gambia	0	0.0%	0	--	5,568	--	-5,568	-56.9%	0	--
Togo	0	0.0%	0	--	0	--	10,584	101.0%	-10,584	-23.2%
Tunisia	0	0.0%	0	--	0	--	0	--	0	--
Uganda	0	0.0%	183	9.6%	1,491	71.3%	102,432	172.2%	-104,106	-57.2%
Western Sahara	12	0.0%	-12	0.0%	0	--	0	--	0	--
Zambia	-26,800	-34.3%	-154,013	-24.7%	118,504	250.5%	62,310	1155.5%	0	--
Zimbabwe	-11,813	-4.9%	-7,144	-5.2%	13,908	103.5%	5,050	--	0	--
**Grand total**	**-463,059**	**-4.4%**	**-609,744**	**-7.7%**	**685,060**	**39.8%**	**1,238,829**	**28.3%**	**-851,087**	**-15.8%**

Values were obtained by subtracting the Eco-climatic index (*EI*) estimates for future *An. gambiae* distribution from projected scenario 2 criteria to the *EI* estimates obtained under current climate for each country. Percentages (%) were calculated by dividing each absolute area change to the current area value per country.

**Table 3:** **Absolute change in areas (km**^**2**^**) for *An. arabiensis *** **per African country under scenario 1**

**Countries**	***EI*** **= 0**		***EI*** **= (0.1-5)**		***EI*** **= (5–10)**		***EI*** **= (10–30)**		***EI*** **= (30–100)**	
	**(km**^**2**^**)**	**%**	**(km**^**2**^**)**	**%**	**(km**^**2**^**)**	**%**	**(km**^**2**^**)**	**%**	**(km**^**2**^**)**	**%**
Algeria	-18,029	-0.9%	18,029	7.0%	0	--	0	--	0	--
Angola	-119	-5.3%	-122,371	-13.6%	42,468	16.3%	80,021	90.1%	0	--
Benin	0	0.0%	9	--	-9	0.0%	0	--	0	--
Botswana	-4,875	-18.7%	-201,103	-64.4%	-102,862	-45.3%	308,841	2144.3%	0	--
Burkina Faso	0	--	0	--	2,463	0.9%	-2,463	-24.0%	0	--
Burundi	0	0.0%	-829	-3.2%	829	54.9%	0	--	0	--
Cameroon	-90,100	-78.7%	91,441	33.5%	-1,342	-1.7%	0	--	0	--
Cape Verde	0	0.0%	0	--	0	--	0	--	0	--
Central African Republic	0	--	62,703	15.6%	-62,703	-28.7%	0	--	0	--
Chad	-148	-4.1%	-38,316	-7.4%	38,464	5.2%	0	--	0	--
Comoros	0	0.0%	0	--	0	0.0%	0	--	0	--
Congo	-107,537	-83.9%	107,537	49.5%	0	--	0	--	0	--
Congo, DRC	-144,492	-49.6%	139,848	6.9%	4,643	53.3%	0	--	0	--
Cote d’Ivoire	-150	-5.5%	-892	-0.5%	1,732	1.2%	-690	-100.0%	0	--
Djibouti	0	0.0%	-4,091	-27.0%	4,091	79.8%	0	--	0	--
Egypt	0	0.0%	0	0.0%	0	--	0	--	0	--
Equatorial Guinea	-1,598	-6.0%	1,598	1943.1%	0	--	0	--	0	--
Eritrea	-824	-28.4%	-17,267	-22.3%	10,546	26.6%	7,545	--	0	--
Ethiopia	-856	-34.4%	-267,550	-31.1%	257,782	97.1%	10,624	153.7%	0	--
Gabon	-60,795	-33.3%	60,795	75.4%	0	--	0	--	0	--
Ghana	0	0.0%	-5,698	-59.8%	14,531	7.8%	-8,833	-19.8%	0	--
Guinea	2,714	109.6%	-2,196	-0.9%	-518	-9.7%	0	--	0	--
Guinea-Bissau	0	0.0%	0	0.0%	0	--	0	--	0	--
Kenya	-1,298	-46.0%	-162,139	-52.5%	57,455	27.5%	105,982	166.2%	0	--
Lesotho	-29,693	-96.4%	29,693	--	0	--	0	--	0	--
Libya	0	0.0%	0	0.0%	0	--	0	--	0	--
Madagascar	-2,126	-21.9%	-19,475	-5.7%	3,924	2.3%	17,677	23.5%	0	--
Malawi	0	--	-2,431	-2.2%	2,431	54.7%	0	--	0	--
Mali	-540	-1.5%	-32,144	-4.8%	39,024	7.2%	-6,340	-100.0%	0	--
Mauritania	-2,013	-1.3%	-864	-0.1%	2,877	1.2%	0	--	0	--
Morocco	0	0.0%	0	--	0	--	0	--	0	--
Mozambique	-92	-2.6%	-124,300	-39.3%	97,249	30.5%	27,143	17.5%	0	--
Namibia	-47,193	-24.1%	-3,795	-1.4%	-49,822	-21.4%	100,810	77.0%	0	--
Niger	-441	-4.4%	-43,709	-6.0%	40,767	9.7%	3,383	14.8%	0	--
Nigeria	1,868	30.4%	26,586	8.4%	-28,454	-4.8%	0	--	0	--
Rwanda	-2	-100.0%	-4,175	-16.8%	4,177	3822.3%	0	--	0	--
Sao Tome & Principe	0	0.0%	0	--	0	--	0	--	0	--
Senegal	0	0.0%	-11,289	-19.4%	11,289	8.1%	0	--	0	--
Seychelles	0	0.0%	0	--	0	--	0	--	0	--
Sierra Leone	1,312	33.9%	-1,312	-1.9%	0	--	0	--	0	--
Somalia	-4,165	-41.7%	-46,632	-9.4%	39,747	45.7%	11,050	24.8%	0	--
South Africa	-320,421	-35.1%	190,047	72.8%	63,538	185.0%	66,837	568.5%	0	--
Sudan	-3,634	-2.9%	-73,782	-9.0%	-149,745	-12.1%	227,160	71.6%	0	--
Swaziland	-27	-33.3%	-2,626	-32.2%	-3,108	-37.9%	5,761	1484.4%	0	--
Tanzania	-1,285	-35.4%	-180,302	-29.5%	139,132	70.0%	42,455	32.7%	0	--
The Gambia	0	0.0%	-5,206	-79.4%	5,206	161.0%	0	--	0	--
Togo	0	0.0%	10,110	--	-8,555	-16.1%	-1,555	-51.3%	0	--
Tunisia	0	0.0%	0	--	0	--	0	--	0	--
Uganda	-187	-100.0%	-21,898	-13.9%	2,798	3.5%	19,287	211.0%	0	--
Western Sahara	-5,159	-4.4%	5,159	3.4%	0	--	0	--	0	--
Zambia	-900	-100.0%	-44,041	-7.0%	44,941	36.8%	0	--	0	--
Zimbabwe	-5,556	-96.0%	-83,346	-37.8%	15,793	11.3%	73,109	289.4%	0	--
**Grand total**	**-847,964**	**-11.3%**	**-780,622**	**-5.6%**	**540,783**	**7.3%**	**1,087,803**	**93.7%**	**0**	**--**

Values obtained by subtracting the Eco-climatic index (*EI*) estimates for future *An. arabiensis* distribution from projected scenario 1 criteria to the *EI* estimates obtained under current climate for each country. Percentages (%) were calculated by dividing each absolute area change to the current area value per country.

**Table 4:** **Absolute change in areas (km**^**2**^**) for *An. arabiensis *** **per African country under scenario 2**

**Countries**	***EI*** **= 0**		***EI*** **= (0.1-5)**		***EI*** **= (5–10)**		***EI*** **= (10–30)**		***EI*** **= (30–100)**	
	**(km**^**2**^**)**	**%**	**(km**^**2**^**)**	**%**	**(km**^**2**^**)**	**%**	**(km**^**2**^**)**	**%**	**(km**^**2**^**)**	**%**
Algeria	-65,468	-3.2%	65,468	25.5%	0	--	0	--	0	--
Angola	6,405	285.3%	14,451	1.6%	12,113	4.6%	-32,969	-37.1%	0	--
Benin	0	0.0%	84,032	--	-84,032	-71.0%	0	--	0	--
Botswana	-23,109	-88.5%	-218,861	-70.1%	-106,614	-47.0%	348,584	2420.2%	0	--
Burkina Faso	0	--	1,794	--	8,479	3.2%	-10,273	-100.0%	0	--
Burundi	0	0.0%	1,510	5.9%	-1,510	-100.0%	0	--	0	--
Cameroon	-92,954	-81.2%	104,920	38.5%	-12,256	-15.5%	290	--	0	--
Cape Verde	0	0.0%	0	--	0	--	0	--	0	--
Central African Republic	1,072	--	145,689	36.3%	-146,761	-67.3%	0	--	0	--
Chad	0	0.0%	-21,437	-4.1%	-9,166	-1.2%	30,603	--	0	--
Comoros	0	0.0%	687	--	-687	-73.9%	0	--	0	--
Congo	-122,043	-95.2%	122,043	56.2%	0	--	0	--	0	--
Congo, DRC	-174,290	-59.8%	182,956	9.0%	-8,666	-99.5%	0	--	0	--
Cote d’Ivoire	143	5.3%	123,466	72.9%	-122,918	-82.9%	-690	-100.0%	0	--
Djibouti	0	0.0%	5,124	33.8%	-5,124	-100.0%	0	--	0	--
Egypt	0	0.0%	0	0.0%	0	--	0	--	0	--
Equatorial Guinea	-26,279	-98.8%	26,221	31886.6%	58	--	0	--	0	--
Eritrea	-589	-20.3%	-12,907	-16.7%	9,293	23.4%	4,203	--	0	--
Ethiopia	-896	-36.0%	39,167	4.6%	-34,069	-12.8%	-4,203	-60.8%	0	--
Gabon	-173,079	-94.9%	168,918	209.6%	4,161	--	0	--	0	--
Ghana	0	0.0%	88,074	924.9%	-44,340	-23.9%	-43,734	-97.8%	0	--
Guinea	14,326	578.3%	-8,996	-3.8%	-5,329	-100.0%	0	--	0	--
Guinea-Bissau	0	0.0%	0	0.0%	0	--	0	--	0	--
Kenya	-473	-16.7%	-57,755	-18.7%	14,451	6.9%	42,727	67.0%	1,050	--
Lesotho	-30,673	-99.6%	29,680	--	886	--	107	--	0	--
Liberia	3,185	134.7%	-3,185	-3.4%	0	--	0	--	0	--
Libya	-1,939	-0.1%	1,939	1.1%	0	--	0	--	0	--
Madagascar	-1,136	-11.7%	25,540	7.5%	-259	-0.2%	-24,145	-32.1%	0	--
Malawi	0	--	3,776	3.3%	-3,776	-85.0%	0	--	0	--
Mali	-14,680	-40.2%	-33,192	-4.9%	54,212	10.0%	-6,340	-100.0%	0	--
Mauritania	-5,726	-3.7%	-52,690	-8.1%	44,558	19.1%	13,859	--	0	--
Morocco	-120,135	-29.6%	120,135	--	0	--	0	--	0	--
Mozambique	-92	-2.6%	22,738	7.2%	25,351	7.9%	-47,997	-31.0%	0	--
Namibia	-91,301	-46.7%	-37,157	-13.9%	25,803	11.1%	102,655	78.4%	0	--
Niger	-7,106	-71.4%	-59,912	-8.2%	89,908	21.4%	-22,890	-100.0%	0	--
Nigeria	12,720	207.2%	211,503	66.7%	-224,224	-38.0%	0	--	0	--
Rwanda	1	31.9%	109	0.4%	-109	-100.0%	0	--	0	--
Sao Tome & Principe	-542	-76.5%	542	--	0	--	0	--	0	--
Senegal	0	0.0%	29,424	50.6%	-29,424	-21.2%	0	--	0	--
Seychelles	0	0.0%	0	--	0	--	0	--	0	--
Sierra Leone	12,880	332.4%	-12,880	-18.6%	0	--	0	--	0	--
Somalia	-6,118	-61.2%	-221,150	-44.6%	175,783	201.9%	51,485	115.5%	0	--
South Africa	-592,894	-64.9%	416,362	159.6%	86,292	251.3%	90,240	767.6%	0	--
Sudan	-1,929	-1.6%	166,575	20.4%	30,088	2.4%	-194,734	-61.4%	0	--
Swaziland	-27	-33.3%	-2,713	-33.2%	-3,715	-45.3%	6,455	1663.2%	0	--
Tanzania	459	12.6%	65,642	10.8%	-1,941	-1.0%	-64,160	-49.5%	0	--
The Gambia	0	0.0%	3,233	49.3%	-3,233	-100.0%	0	--	0	--
Togo	0	0.0%	45,722	--	-42,690	-80.4%	-3,032	-100.0%	0	--
Tunisia	0	0.0%	0	--	0	--	0	--	0	--
Uganda	280	149.7%	68,589	43.6%	-59,762	-75.7%	-9,107	-99.6%	0	--
Western Sahara	-7,146	-6.1%	7,146	4.7%	0	--	0	--	0	--
Zambia	-900	-100.0%	31,898	5.1%	-30,998	-25.4%	0	--	0	--
Zimbabwe	-5,439	-93.9%	-11,414	-5.2%	-5,354	-3.8%	22,207	87.9%	0	--
**Grand total**	**-1,515,491**	**-20.2%**	**1,670,823**	**12.0%**	**-405,522**	**-5.5%**	**249,141**	**21.5%**	**1,050**	**--**

Values were obtained by subtracting the Eco-climatic index (*EI*) estimates for future *A. arabiensis*distribution from projected scenario 2 criteria to the *EI* estimates obtained under current climate for each country. The percentages (%) were calculated by dividing each absolute area change to the current area value per country. Tables 1, 2, 3, 4: (--) Represents the absolute change within a scale range in which the current value of area was zero (0); (+) indicates an increase; (-) indicates a reduction in areas; (0) means no change.

For *An. gambiae*, and with the two projected scenarios, Classes 1 and 2 display similar trend of the results with good number of the countries having either, 0 or negative area (Tables 1 and 2). In Class 1, a possible loss in area of 1,678,200 km^2^ (Table 1) and 463,059 km^2^ (Table 2) for projected scenarios 1 and 2 are predicted, respectively. Class 2 showed an analogous tendency with an estimated reduction in area of 150,534 km^2^ (Table 1) and 609,744 (Table 2) under succeeding scenarios. These losses of area in the classes express the reduction in zones of little and no suitability for the species. In contrast, a possible increment of 698,477 km^2^, 378,946 km^2^, 751,311 km^2^ (Table 1) is predicted under scenario 1 for Classes 3, 4 and 5, respectively. Under scenario 2, a similar tendency occurs in Class 3 (685,060 km^2^) and Class 4 (1,238,829 km^2^). An approximation of 851,087 km^2^ reduction in area is estimated for Class 5 under scenario 2. Overall, the results show that if the climate changes according to scenario 1, *An. gambiae* will most likely expand its range from zones of no and low suitability into zones of sequential; high risk of permanent establishment and long-term survival. The African countries to be affected under this scenario include Angola, Burundi, Comoros, Ethiopia, Kenya, Malawi, Mali, South Africa, Tanzania and Zambia (Table 1). Under climate change scenario 2, *An. gambiae* will possibly concentrate its distribution in areas with sequential and high risk of permanent establishment. Angola, Cameroon, Ethiopia, Guinea, Mozambique, Niger, Sierra Leone, South Africa, Uganda, Zambia and Zimbabwe are the countries predicted to be prone to a 50% total country area shift from no and low survival into areas of sequential and high risk of permanent establishment of *An. gambiae* (Table 2)*.*

Results for *An. arabiensis* under projected scenarios 1 and 2 are presented in Tables 3 and 4, respectively. Under the first climate change scenario (Table 3), area losses of 847,964 km^2^ (Class 1) and 780,622 (Class 2) were estimated. Further, area increments of 540,783 km^2^ and 1,087,803 km^2^ were projected for Classes 3 and 4, respectively with no evident change of area for class 5. *Anopheles arabiensis* displays a considerable loss of area equal to 1,515,491 km^2^ (Class 1) and a gain of 1,670,823 km^2^ (C 2) with scenario 2. Within this scenario, an additional loss of 405,522 km^2^ may occur in Class 3 and increase in two areas evaluated at 249,141 km^2^ (Class 4) and at 1,050 km^2^ (Class 5). From these results, *An. arabiensis* will most likely be found in Angola, Botswana, Burundi, Congo DRC, Djibouti, Ethiopia, Kenya, Malawi, Namibia, Rwanda, South Africa, Sudan, Swaziland, The Gambia, Uganda, and Zimbabwe (Table 3). Area increases were predicted in these countries belonging to Classes 3 and 4 under climate change scenario 1. In contrast with climate change scenario 2, majority of area shifts will converge in zones with very little suitability for survival of *An. Arabiensis* (Table 4).

**Figure 3:**
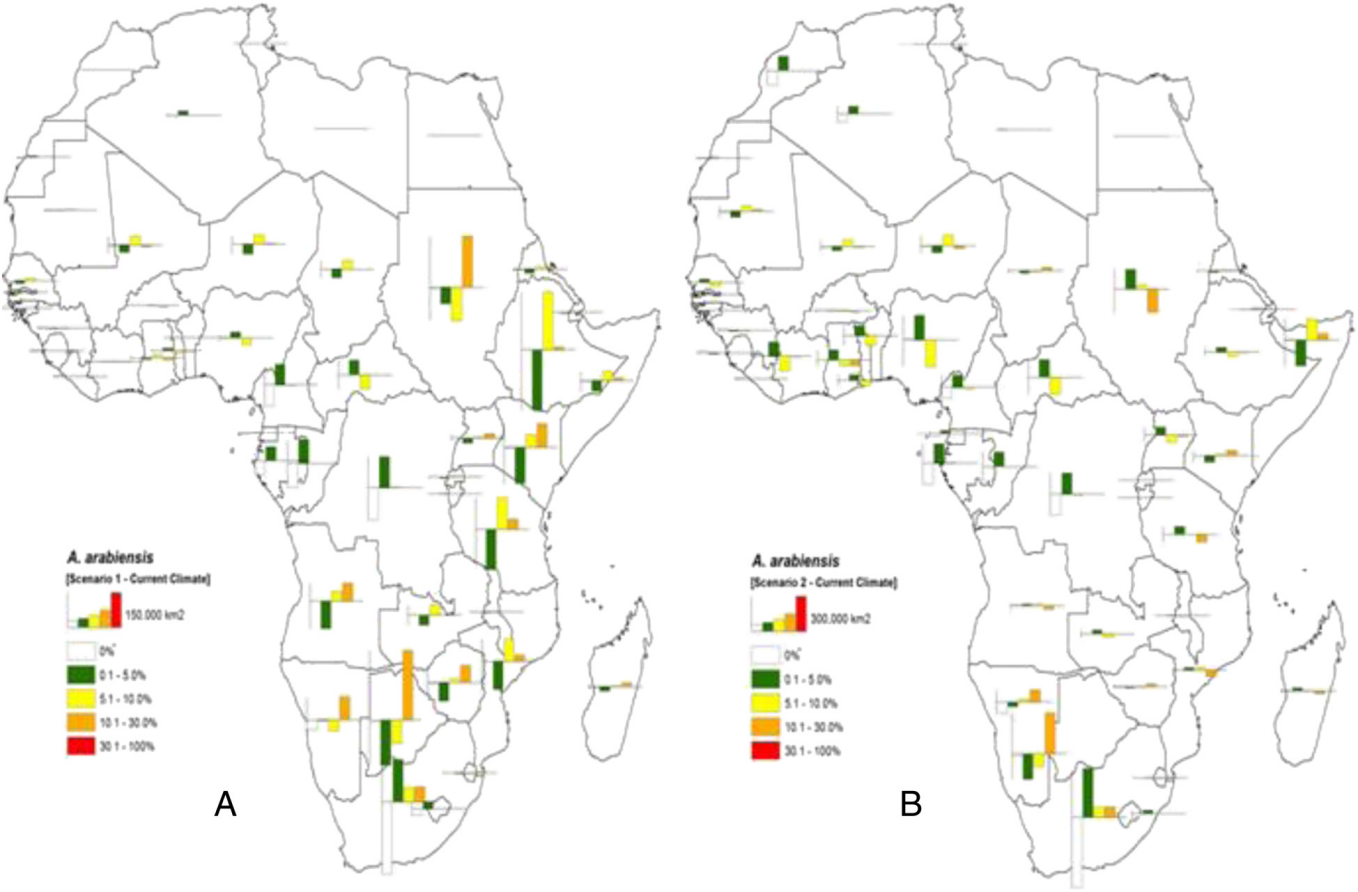
**Bar charts showing the absolute change in areas (km**^**2**^**) that may occur due to possible change in climate for *Anopheles arabiensis *per African country.** Values obtained by subtracting the Eco-climatic index (*EI*) estimates of projected scenarios 1 **(A)** and 2 **(B)** criteria to the *EI* estimates obtained under current climate for each country. The height of a bar shows the proportion of area in a scale of **(A)** 150,000 km^2^ and **(B)** 130,000 km^2^; bar display on the positive axis represents an increasing area and a reverse bar in the negative axis, a decreasing area.

Figures 2 and 3 represent the summary of absolute change in area per country for *An. gambiae* and *An. arabiensis*, respectively. A bar graph was used to illustrate visually and display the amounts of absolute change of area. The display allows us to compare levels of suitability for the *Anopheles* species, and to quickly make generalizations about possible change of ranges with changing climate. The height of a bar indicates the proportion of area that is increasing or decreasing. A bar displayed on the positive plane of the axes represents an increasing area where as a reverse bar represents a decreasing area. Such quantitative chart maps are useful for decision-making, they easily guide on the identification of countries with high risk of permanent establishment of malaria vectors due to potential change in climate. Overall, the proportion of areas that is increasing or decreasing in level of survival for malaria vectors is more pronounced in eastern and southern African countries than in western African countries. Angola, Ethiopia, Kenya, Mozambique, Tanzania, South Africa and Zambia appear most likely to be affected in terms of absolute change of malaria vectors suitability zones under the selected climate change scenarios.

## Discussion

The importance of projecting the future spread of malaria under various climate change scenarios cannot be overemphasized as it helps policy-makers to identify vulnerable communities and to better manage malaria epidemics. In such projections, various climate models have been applied producing different climate changes and correspondingly shift the future distribution and variability of malaria transmission. However, planning and adapting to the projected boundary shifts of malaria vectors under changing climate, necessitates innovative approaches.

We provide an improved measure of malaria risk projections by defining geographical locations favourable for the malaria vectors establishment and survival. We define areas likely to become more suitable for transmission of malaria owing to suitable areas for the vectors promoting parasite survival and transmission by vector. Survival of the vectors from year-to-year is a prerequisite for malaria endemicity and remains a key component in the epidemiology and spread of the disease. Our predictions take into account the differential impact of climate change on the dynamics of these key malaria vectors as dictated by their individual dynamics and life cycle parameters which obviously impart differently on the patterns and spread of malaria.

Within the African setting, the suitability for malaria transmission has been projected to change by varying degrees and directions over much of the Sahel, eastern and southern Africa [9,11]. The population at risk has been projected to decrease across West Africa and the Sahel because of a drier climate, [6,18] but to increase in both East and South Africa [6,26]. In the same light, an earlier study [14] predicted likely shifts in the boundaries of *An. gambiae* and *An. arabiensis* southwards and eastwards of Africa, respectively, rather than jumps into quite different climatic environments. Animated by the need for local level-based malaria risk projections, this study represents advancement in our ability to predict future malaria trends especially at the country level. Our results illustrate the amounts of absolute change of area per country that allows us to compare levels of suitability for the *Anopheles* species, and to quickly make generalizations about possible change of ranges with changing climate.

Predicted areas in terms of absolute change for suitability or establishment of both vectors vary under the two climate scenarios (1 and 2) considered. This finding indicates that the susceptibility in their distribution is dictated by variable climatic patterns notably temperature, rainfall and humidity. These species are known to live in sympatry although adaptive preference in ecology for these species has been highlighted with *An. gambiae* preferring wet and humid zones, and *An. arabiensis* drier climates [27]. An added biological complexity is that there are distinct chromosomal forms of *An. gambiae* with distinct climatic preferences [28] although there is not enough available data to separately map these forms. Given that the greatest effect of climate change on transmission is likely to be observed at the extremes of temperature ranges [3], it was surprising that under scenario 1, adaptation to areas of long term survival (class 5) was evident for *An. gambiae* but not for *An. arabiensis*. Therefore, the combined effect of temporal and spatial changes in temperature, precipitation and humidity are expected to occur under different climate change scenarios which will affect the biology and ecology of vectors and intermediate hosts and consequently the risk of disease transmission [3]. The changed area of establishment is likely to be affected by intensity of vector control efforts and health infrastructure. For example, following long term use of IRS and ITNs, there has been a proportionate increase in *An. arabiensis* compared to its closely related sibling species *An. gambiae* s.s in East Africa [29,30], as this species is less susceptible to the indoor control strategies because of its plastic and more exophilic tendencies.

We have presented a country-based projection of malaria transmission under different climate change scenarios with key parameters –temperature, rainfall and humidity. Although we do not assert that climate alone is the main determinant factor for *Anopheles* species existence, it is the only factor with available country data that provides an initial estimate of the potential range of *Anopheles* species. The present study should rather be seen as an illustration of the substantial influence, which the direct effects of climate change may represent to malaria vectors redistribution within each African country. The results presented here should be interpreted as an indication of the sensitivity of malaria vectors to climatic changes, particularly temperature rise. More elaborate future risk assessments of climate change on malaria vectors in Africa will ultimately need to include the ecology and behavior of the host and vector, non-climatic factors such as gross domestic product (GDP), future population growth and migration, socioeconomic status, urbanization and malaria control status and vector in each country.

### Recommendations

The overall outcome of the study has led to recommendations in form of best practices which if put in place could help in preventing malaria under a changing climate context. The proposed guidelines highlight potential actions and suggestions for mitigating the negative impact of climate change on malaria risk in Africa, which can be extended to other vector borne diseases.

### Technical innovation

A combined application of computing technology, telephone networks, geographical information system (GIS) and other technologies can be invaluable in the development of computerized tools for use at local, regional and continental levels for effective management of malaria. These tools include relevant literature, decision support software, and access to one or more central databases of *Anopheles* biology, ecology, population densities and related climate information in a GIS database for adequate monitoring of the species shifts [31-36]. Such innovative tools can be used to alert officials on impending outbreaks days or months ahead. Innovative investigations could also be oriented towards developing suitable field tools for monitoring essential characteristics of vector populations for improved understanding of their adaptive capacity with changing climate.

### Strengthening internal and cross-border vector surveillance

A step-by-step approach to a regional framework for implementing integrated malaria vector management from the district to regional and Africa wide level is needed [31]. It is essential that countries and regional blocks in Africa establish appropriate plans for deploying an optimum mix of interventions measures. Such a framework will be helpful in guiding member countries in selectively applying the various available vector control tools based on the epidemiological situation, vector bionomics and behavior, and the socio-behavioral characteristics of the community. This might enhance malaria surveillance within a country and in regions and help in case of urgent need for action, to contain the spread of the vectors. Through regular exchange forums and technical consultations it should be possible to identify, share and disseminate best practices in malaria control including the development of projects with cross-border activities to enhance integration and help in developing a strong Africa union.

### Research

Research is important in understanding and implementing adaptive measures to climate change in Africa. The knowledge gained through research will contribute in laying a foundation for future planning. An investment by African governments and industries in science and technology development is deemed necessary in order to fulfill their pledges [32]. Potential research areas of focus will include an understanding of: 1) the dynamics of malaria transmission by vector populations and the risk of exposure for human populations in various ecosystems; 2) the factors affecting *Anopheles* larval survival and mechanisms controlling adult production in aquatic habitats, and 3) the selective forces that cause some mosquitoes like *An. gambiae* to specialize on humans.

### Enhancing information management and knowledge sharing

Information, education and communication are vital means for improving adaptation to *Anopheles* control; in which case, increased awareness for generations to begin coping with climate change is essential. Teaching the science of climate change in schools may be instituted with a focus on practical skills for the management of climate risks. Dissemination can be facilitated through the media and information tools such as television programmes, short thematic films, brochures, booklets. Special education and training needs may be regularly organized with emphasis on enhancing awareness, stimulating acceptance and active participation of the communities.

## Conclusion

This paper estimates in Km^2^ and for each African country the absolute change in zone of suitability of the major malaria vectors, *An. gambiae* and *An. arabiensis*, under different projected climate scenarios. The potential shifts will have implications for the overall number of people exposed to the malaria vectors and the recrudescence of malaria is likely to be recorded in several new areas and regions. Based on the results, there is a need for developing, compiling and sharing malaria preventive measures, which can be adapted to different climatic scenarios.

## Competing interests

The authors declare that they have no competing interests.

## Authors’ contributions

Conceived and designed the experiments: HEZT. Analyzed the data: HEZT HSJ. Wrote the paper: HEZT DPT HSJ LK IRFD. All authors approved the final version of the manuscript for submission.

## References

[B1] Patz JA Khaliq M Global climate change and health: challenges for future practitioners JAMA 2002 287 2283 2284 10.1001/jama.287.17.2283-JMS0501-3-1 11980532

[B2] McMichael AJ Woodruff RE Hales S Climate change and human health: present and future risks Lancet 2006 367 859 869 10.1016/S0140-6736(06)68079-3 16530580

[B3] Githeko AK Lindsay SW Confalonieri UE Patz JA Climate change and vector-borne diseases: a regional analysis Bull World Health Organ 2000 78 1136 1147 11019462 PMC2560843

[B4] Sutherst RW Global change and human vulnerability to vector-borne diseases Clin Microbiol Rev 2004 17 136 173 10.1128/CMR.17.1.136-173.2004 14726459 PMC321469

[B5] Reiter P Climate change and mosquito-borne disease: knowing the horse before hitching the cart Rev sci tech Off int Epiz 2008 27 2 383 398 18819667

[B6] Peterson AT Shifting suitability for malaria vectors across Africa with warming Climates BMC Infect Dis 2009 9 59 10.1186/1471-2334-9-59 19426558 PMC2694813

[B7] Stockwell DRB Peters DP The GARP modelling system: Problems and solutions to automated spatial prediction Int J Geogr Inf Sci 1999 13 143 158 10.1080/136588199241391

[B8] Lafferty K The ecology of climate change and infectious diseases Ecology 2009 90 888 900 10.1890/08-0079.1 19449681

[B9] Lindsay SW Parson L Thomas CJ Mapping the ranges and relative abundance of the two principal African malaria vectors, *Anopheles gambiae* sensu stricto and *An. arabiensis*, using climate data Lond Ser B-Biol Sci 1998 265 847 854 10.1098/rspb.1998.0369 PMC1689061 9633110

[B10] Smith DL McKenzie FE Statics and dynamics of malaria infection in *Anopheles* mosquitoes Malaria J 2004 3 13 10.1186/1475-2875-3-13 PMC449722 15180900

[B11] Levine RS Peterson AT Benedict MQ Geographic and ecologic distributions of the *Anopheles gambiae* complex predicted using a genetic algorithm Am J Trop Med Hyg 2004 70 105 109 14993618

[B12] Pascual M Ahumada JA Chabes LF Rodo X Bouma M Malaria resurgence in the East African highlands: temperature trends revisited Proc Natl Acad Sci U S A 2006 103 5829 5834 10.1073/pnas.0508929103 16571662 PMC1416896

[B13] Dillon ME Wang G Huey RB Global metabolic impacts of recent climate warming Nature 2010 467 704 706 10.1038/nature09407 20930843

[B14] Tonnang ZEH Kangalawe YMR Yanda ZP Predicting and mapping malaria under climate change scenarios: the potential redistribution of malaria vectors in Africa Malaria J 2010 9 111 10.1186/1475-2875-9-111 PMC2873524 20416059

[B15] Martens WJ Niessen LW Rotmans J Jetten TH McMichael AJ Potential impact of global climate change on malaria risk Environ Health Persp 1995 103 458 464 10.1289/ehp.95103458 PMC1523278 7656875

[B16] van Lieshout M Kovats RS Livermore MTJ Martens P Climate change and malaria: analysis of the SRES climate and socio-economic scenarios Global Environ Chang 2004 14 87 99 10.1016/j.gloenvcha.2003.10.009

[B17] Ermert V Fink AH Morse AP Paeth H The impact of regional climate change on malaria risk due to greenhouse forcing and land-use changes in tropical Africa Environ Health Perspect 2012 120 77 84 21900078 10.1289/ehp.1103681 PMC3261943

[B18] Tanser FC Sharp B le Sueur D Potential effect of climate change on malaria transmission in Africa Lancet 2003 362 1792 1798 10.1016/S0140-6736(03)14898-2 14654317

[B19] Bouma MJ Baeza A terVeen A Pascual M Global malaria maps and climate change: a focus on East African highlands Trends Parasitol 2011 27 10 421 422 10.1016/j.pt.2011.07.003 21873114

[B20] Mapping Malaria Risk in Africa 2010 [http://www.mara.org.za/]

[B21] Koum G Yekel A Ndifon B Simard F Design and implementation of a mosquito database through an entomological ontology Bioinform 2005 21 2797 2802 10.1093/bioinformatics/bti399 15981318

[B22] Moffett A Strutz S Guda N González C Ferro MC Cordero VS Sarkar S A global public database of disease vector and reservoir distributions PLoS Negl Trop Dis 2009 3 3 10.1371/journal.pntd.0000378 PMC2656641 19333367

[B23] Sutherst RW Maywald GF A computerised system for matching climates in ecology Agric Ecosyst Environ 1985 13 281 299 10.1016/0167-8809(85)90016-7

[B24] Intergovernmental Panel on Climate Chang (IPCC) Houghton JT, Ding Y, Griggs DJ, Noguer M, van der Linden PJ, Dai X, Maskell K, Johnson CA Climate Change: The Scientific Basis. Contribution of Working Group I to the Third Assessment Report of the Intergovernmental Panel on Climate Change 2001 Cambridge, United Kingdom and New York, NY, USA: Cambridge University Press 881

[B25] Roshan JV Kang L Regression-based inverse distance weighting with applications to computer experiments Technometrics 2011 53 3 254 265 10.1198/TECH.2011.09154

[B26] Thomas CJ Davies G Dunn CE Mixed picture for changes in stable malaria distribution with future climate in Africa Trends Parasitol 2004 20 216 220 10.1016/j.pt.2004.03.001 15105021

[B27] Coluzzi M Sabatini A Petrarca V di Deco MA Chromosomal differentiation and adaptation to human environments in the *Anopheles gambiae* complex T Roy Soc Trop Med H 1979 73 483 497 10.1016/0035-9203(79)90036-1 394408

[B28] Touré YT Petrarca V Traoré SF Coulibaly A Maiga HM Sankaré O Sow M Di Deco MA Coluzzi M The distribution and inversion polymorphism of chromosomally recognized taxa of the *Anopheles gambiae* complex in Mali, West Africa Parassitologia 1998 40 477 511 10645562

[B29] Bayoh MN Mathias DK Odiere MR Mutuku FM Kamau L Gimnig JE Vulule JM Hawley WA Hamel MJ Walker ED *Anopheles gambiae*: historical population decline associated with regional distribution of insecticide-treated bed nets in western Nyanza Province, Kenya Malaria J 2010 9 62 10.1186/1475-2875-9-62 PMC2838909 20187956

[B30] Russell TL Lwetoijera DW Maliti D Chipwaza B Kihonda J Charlwood JD Smith TA Lengeler C Mwanyangala MA Nathan R Knols BG Takken W Killeen GF Impact of promoting longer-lasting insecticide treatment of bed nets upon malaria transmission in a rural Tanzanian setting with pre-existing high coverage of untreated nets Malaria J 2010 9 187 10.1186/1475-2875-9-187 PMC2902500 20579399

[B31] World Health Organization Mathematical Modelling to Support Malaria Control and Elimination Roll Back Malar Prog Impact Ser 2010 5 1 48

[B32] African Press Organization Science, technology, innovation and capacity building for addressing climate change Issues Pap 2010 10 1 10

[B33] Lambin EF Tran A Vanwambeke SO Linard C Soti V Pathogenic landscapes: interactions between land, people, disease vectors, and their animal hosts Int J Health Geogr 2010 9 54 10.1186/1476-072X-9-54 20979609 PMC2984574

[B34] Foley DH Wilkerson RC Birney I Harrison S Christensen J Christensen J Rueda LM MosquitoMap and the Mal-area calculator: new web tools to relate mosquito species distribution with vector borne disease Int J Health Geogr 2010 9 11 10.1186/1476-072X-9-11 20167090 PMC2837623

[B35] Linard C Tatem AJ Large-scale spatial population databases in infectious disease research Int J Health Geogr 2012 11 7 10.1186/1476-072X-11-7 22433126 PMC3331802

[B36] Dambach P Machault P Lacaux JP Vignolles C Sié A Sauerborn R Utilization of combined remote sensing techniques to detect environmental variables influencing malaria vector densities in rural West Africa Int J Health Geogr 2012 11 8 10.1186/1476-072X-11-8 22443452 PMC3331805

